# The Viral AlphaFold Database of monomers and homodimers reveals conserved protein folds in viruses of bacteria, archaea, and eukaryotes

**DOI:** 10.1126/sciadv.adz8560

**Published:** 2025-10-01

**Authors:** Roni Odai, Michèle Leemann, Tamim Al-Murad, Minhal Abdullah, Lena Shyrokova, Tanel Tenson, Vasili Hauryliuk, Janani Durairaj, Joana Pereira, Gemma C. Atkinson

**Affiliations:** ^1^ Department of Experimental Medical Science, Lund University, Lund, Sweden.; ^2^ Biozentrum, University of Basel, Basel, Switzerland.; ^3^ Institute of Technology, University of Tartu, Tartu, Estonia.; ^4^ Science for Life Laboratory, Lund, Sweden.; ^5^ NanoLund, Lund University, Lund, Sweden.; ^6^ Lund University Virus Centre, Lund, Sweden.; ^7^ SIB Swiss Institute of Bioinformatics, Basel, Switzerland.

## Abstract

Viruses are the most abundant and genetically diverse entities on Earth, yet the functions and evolution of most viral proteins remain poorly understood. Their rapid evolution often obscures evolutionary relationships, limiting the ability to assign functions using sequence-based methods. Although the conservation of protein fold can reveal deep homologies, viral proteins remain underrepresented in structural databases. We address this by clustering viral sequences from RefSeq and predicting the structures of ~27,000 representative proteins using AlphaFold2 to create the Viral AlphaFold Database (VAD). We uncover conserved folds in diverse viruses infecting bacteria, archaea, and eukaryotes. We predict homodimers and make comparisons to the Protein Data Bank, providing data on oligomerization potential. We reveal considerable functional darkness in the viral protein universe and report the discovery and validation of an uncharacterized toxin-antitoxin system. The VAD provides a foundation for exploring viral structure-function relationships, including ancient folds shaping viral interactions across all life.

## Teaser

VAD is a Viral AlphaFold Database of protein monomers and homodimers from viruses infecting hosts across the tree of life.

## INTRODUCTION

Modern deep learning structural prediction methods, such as AlphaFold and RosettaFold, have revolutionized the ability to use structural information in research (*1*–*3*). As well as the use of inferred folds to make predictions about the function that can be tested experimentally, structure versus structure comparison methods allow the discovery of homology that is not detectable at the sequence level using tools such as Foldseek (*4*) and distance-matrix alignment (Dali) (*5*). This can be especially informative for proteins of viruses, which evolve fast because of short generation times, large population sizes, high mutation rates, and host-virus arms races (*6*, *7*). The taxonomic domain of viruses includes both eukaryote-infecting viruses and bacteriophages that infect bacteria and archaea. Despite the fundamental differences between their hosts—including cell structure, gene regulation, and evolutionary history—viruses across these domains can share conserved protein folds. The single jellyroll is one of the most common capsid folds and can be found in various phages and eukaryote-infecting viruses (*8*), while the capsid protein of bacteriophage HK97 has homologs in herpes viruses, adenoviruses, and viruses of archaea and protists (*8*, *9*). Most recently, phosphodiesterases of both phages and eukaryote-infecting viruses have been found to contain the same fold and been experimentally confirmed to have a shared role in immune evasion (*10*, *11*).

The ability to search for homologous proteins and infer the function at the fold level depends upon the availability of large and diverse protein structure databases. The Protein Data Bank (PDB) has been an essential resource for structural comparisons for decades, storing and organizing experimentally determined structures of proteins and complexes (*12*). A massive increase in the numbers and diversity of publicly available structures came with the release of the EBI AlphaFold database (AFDB) (*13*), which includes predicted structures for most proteins in the UniProt database, vastly expanding the number of available protein structures beyond what is present in the PDB. The structural comparison of these millions of protein folds has enabled the construction of networks of structural communities, annotated with their functional “brightness” or “darkness,” depending on how well annotated the biochemical and biological functions of those proteins are (*14*).

A substantial portion of the known protein fold universe remains functionally dark, and this is likely an underestimate of the true extent of structural and functional dark matter. One group that remains underexplored in structural biology is viruses. Despite being regarded as the most abundant biological entity on Earth and being an immense reservoir of genetic diversity (*15*, *16*), viruses have so far been excluded from the EBI AFDB. This is primarily due to the problem of polyproteins. Polyproteins are composed of multiple viral peptides that are translated as a single concatenated peptide and subsequently proteolytically cleaved into individual mature proteins using viral or host proteases (*17*, *18*). While these polyprotein structures complicate structural prediction, the potential benefits of uncovering viral protein structural diversity outweigh these challenges. Recent viral structure databases, such as the BFVD (*19*) and Nomburg24 (*10*) projects, have addressed this gap to a large extent. However, these resources rely on less accurate methods than the reference AlphaFold2 implementation and are limited to monomeric structural predictions.

Here, we have clustered 647,000 unique viral sequences from the National Center for Biotechnology Information (NCBI) RefSeq database (*20*), predicted the structure of ~27,000 representatives using AlphaFold2 (*1*), and predicted higher-order oligomeric states where possible. This represents a high-quality resource for exploring viral protein diversity, including in the context of host taxonomy. We have identified 1142 clusters of viral proteins that share the same fold across viruses infecting two or more domains of cellular life (bacteria, eukaryotes, or archaea). We have characterized the functional darkness of viral folds, predicted homodimerization tendencies and structures for almost all proteins in our database, and predicted higher-order multimeric states where possible. Through searching for functionally dark protein structures in conserved neighborhoods, we have uncovered and experimentally validated a previously unidentified type II toxin-antitoxin (TA) system, KreTA, found in prophages of bacteria and mapped conserved and variable folds across virus-host interactions, including defense and antidefense systems. All structures are available at data-sharing.atkinson-lab.com/vad/, and the structural clusters can be searched and explored at vad.atkinson-lab.com.

## RESULTS

### Clustering the diversity of viral proteins at the sequence level reveals homology among proteins from viruses that infect hosts in different domains of life

**Fig. 1.**
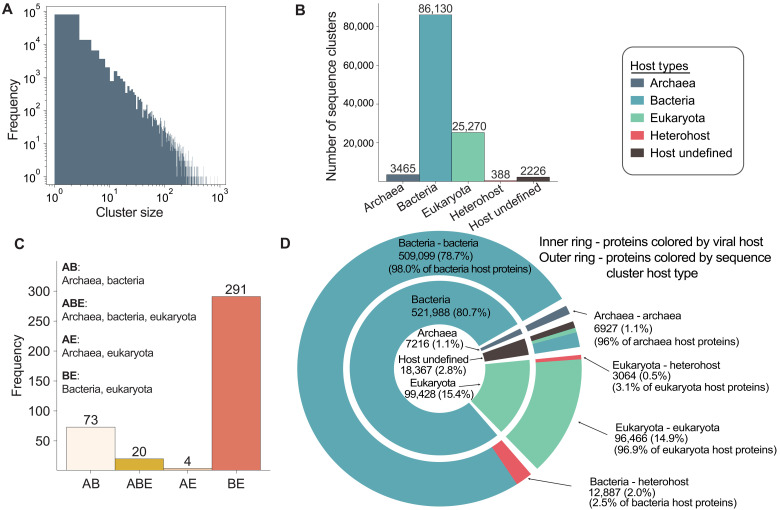
Sequence clustering and host-type assignment of viral proteins and clusters. (**A**) Distribution of sequence clusters by size. (**B**) Distribution of sequence clusters by host type. (**C**) Distribution of sequence heterohost clusters based on host domain composition. (**D**) Distribution of proteins by viral host (inner ring) and sequence cluster host type (outer ring), where viral host segments correspond to cluster type segments, displaying the cluster host-type membership of proteins by viral host.

Because of the computational challenges of predicting structures for all available viral protein sequences, we aimed to assemble and fold a representative subset that captures broad diversity across the sequence space. To do this, we first downloaded protein sequences from the NCBI RefSeq database (*20*), limiting by taxonomy to virus (NCBI taxonomy ID 10239). The taxonomic distribution of these 647,000 sequences is depicted in fig. S1, spanning the 10 most abundant viral taxa across the kingdom, phylum, and family levels. The host of each virus was assigned by querying the Virus-Host database (*21*). Protein sequences were then clustered using MMseqs2 (*22*), with relatively lenient identity and coverage thresholds (30% and 0.01, respectively) to minimize the number of clusters while maintaining sufficient separation between them (Fig. 1A). This clustering process yielded 117,479 clusters and 61,868 singletons, with an average cluster size of 5.5 members (table S1 and dataset S1).

The assignments from the Virus-Host database (*21*) allowed us to group proteins by host domain (bacteria, archaea or eukaryotes), where a cluster with multiple hosts associated with the proteins within is referred to as a heterohost cluster. It is important to note that this does not imply that the same protein can be found in viruses infecting vastly different hosts; rather, the cluster is composed of homologous proteins that can be found in different host-infecting viruses. Therefore, heterohost cluster composition reflects molecular patterns that are common across viral life. Reflecting the original taxonomic distributions, most clusters comprise proteins from viruses infecting bacteria (Fig. 1B). In total, 388 clusters were assigned a sequence-level heterohost label, most of which comprise proteins from viruses with either bacterial or eukaryotic hosts (Fig. 1C). The distribution of host domain (eukaryota, bacteria, or archaea) before and after clustering is shown in Fig. 1D. To reduce ambiguity, we excluded from these counts any viruses annotated as infecting other viruses, such as virophages that parasitize giant viruses and phage satellites (*23*). Most unique viral protein hosts are bacteria (79.9%), followed by eukaryotes (15.4%). Archaeal hosts are assigned for only 1.1% of the proteins (Fig. 1D).

While some of the sequence-level heterohost clusters contain proteins annotated as structural proteins, i.e., tail fibers, capsids, or their chaperones, most heterohost clusters represent proteins that are annotated to interact with or modify nucleotides or nucleic acids (dataset S1). The most prevalent are proteins involved in replication, nucleases, nucleotide hydrolases, nucleotide modification enzymes, helicases, nucleic acid ligases, and methyltransferases. The largest heterohost sequence cluster (567 proteins, found in viruses infecting either bacteria or eukaryotes) contains homologs of cytidine and deoxycytidylate deaminase—an enzyme that viruses can use to modulate nucleotide pools to favor their own replication (*24*) and that sometimes serves as a component of antiphage defense (*25*). The second biggest sequence cluster contains proteins homologous to the clamp loader of DNA polymerase, which enables efficient and processive replication and is found in bacteriophages and viruses of protists. Last, there are 20 sequence clusters spanning bacteria, eukaryote, and archaeal hosts. The largest of these contains DNA methyltransferases (dataset S1). Thus, at the sequence level, conserved nucleotide-processing functions are often the most readily identifiable across host domains.

### Prediction of representative protein structures to create the VAD

Representatives from the viral protein sequence clusters were folded with AlphaFold2 (*1*), limiting to clusters with a size of 5 or greater. To sample sequence diversity in smaller clusters, we also randomly selected a set of 7243 sequence clusters, with an average cluster size of 1.6. We did not set a strict amino acid length limit but rather folded all proteins that we could, given our computational resources. Compared to the two other predicted structure databases for viral proteins, BFVD (*19*) and Nomburg24 (*10*), our length distribution is most similar to BFVD, with an average length of 176 amino acids (fig. S2). The length distribution in our database has a longer tail because of not using a length cutoff; our largest predicted fold is from a 3595 amino acid–long protein. Nomburg24 predictions tend to be longer on average, probably due to eukaryote-infecting viruses having longer proteins on average than phages. The final Viral AlphaFold Database (VAD) structure dataset consists of 26,962 predictions for monomers and 26,754 homodimer cofolding attempts (of which only a subset is confident homodimers; see below) (dataset S1). The taxonomic distribution of VAD is depicted in fig. S3 (full lineage information is found in dataset S1) and covers the full diversity of viral kingdoms, phyla, and families seen in the starting sequence set (figs. S1 and S3).

**Fig. 2.**
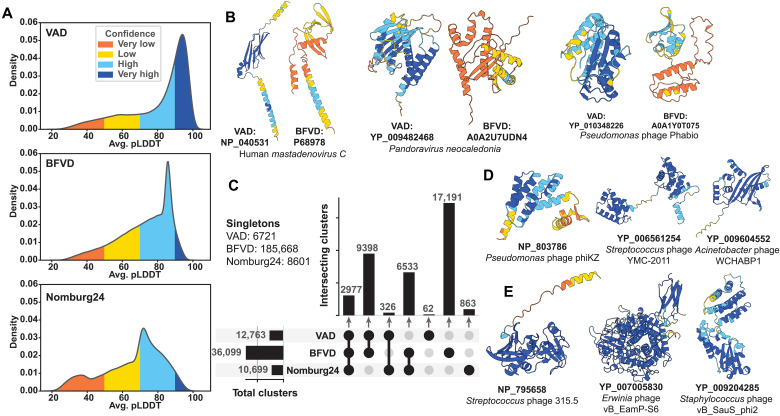
VAD comparison with other databases of viral predicted structures. (**A**) Density plot of average pLDDT distributions for the VAD, BFVD, and Nomburg24. pLDDT is averaged per prediction in each dataset. (**B**) Three examples where the VAD structure pLDDT is higher than the corresponding BFVD structure pLDDT. (**C**) UpSet plot detailing overlaps of nonsingleton structural clusters (with Foldseek easy-cluster coverage of 0.7 and default parameters) across the VAD, BFVD, and Nomburg24 with singleton cluster counts listed. (**D**) Three examples of VAD cluster singletons with >80 pLDDT. (**E**) Three examples of clusters unique to the VAD with >80 pLDDT.

The quality of the predictions in the dataset is assessed with the predicted local difference test (pLDDT) metric (*1*). The VAD dataset has a global average pLDDT of 78, with most predictions reaching an average pLDDT of ~90, reflecting high overall model confidence (Fig. 2A). Compared to Nomburg24 (*10*) and BFVD (*19*), the VAD demonstrates improvements in prediction quality. Among near-identical proteins (>90% sequence identity and coverage) shared between the VAD and BFVD, 74% of VAD models have higher average pLDDT scores, and 54% show an increase of more than 5 units. This highlights the accuracy gains of the standard AlphaFold2 pipeline, with differences in prediction quality likely attributable to BFVD’s use of faster, but potentially less sensitive, multiple sequence alignment construction approaches. Examples of such cases are shown in Fig. 2B.

Structure-based all-versus-all searches using the ~27,000 VAD cluster proteins identify more than twice as many unique cluster-to-cluster relationships as sequence-based searches can find across all 647,000 viral proteins. This indicates that this relatively small, structure-representative set effectively captures relationships within the viral NCBI RefSeq database. The VAD is structurally diverse, as seen by the 12,763 structural clusters and 6721 singletons encompassing the 26,962 VAD cluster proteins (Fig. 2C). In addition, despite its smaller size, the VAD dataset spans a substantial portion of the nonsingleton structural clusters found in both BFVD and Nomburg24, which contain 2.5× and 13× more structures, respectively (Fig. 2C). Examples of singletons and unique VAD clusters are shown in Fig. 2 (D and E, respectively). Together, these results show that the VAD dataset both is structurally diverse and has high confidence, making it a valuable resource for structural searches and for advancing our understanding of viral protein function and evolution.

### Proteins with the same structural fold in the VAD are found in viruses infecting bacteria, archaea, and eukaryotes

Sequence clustering shows that the vast majority of cluster representatives comes from viruses infecting bacterial hosts (Fig. 1B). To test whether structure-based clustering reveals broader relationships across host types, we examined whether proteins from viruses infecting different domains of life share similar folds even when their sequences diverge. We therefore clustered the 26,962 structures in the VAD using Foldseek (*4*) in TMalign mode, which groups proteins by structural similarity. This resulted in 12,894 clusters with 9753 singletons, with an average cluster size of 2.1 (table S2 and dataset S1). Host domains (bacteria, archaea, or eukaryotes) were then assigned to structural clusters using the same host annotations and criteria as for sequence clusters.

**Fig. 3.**
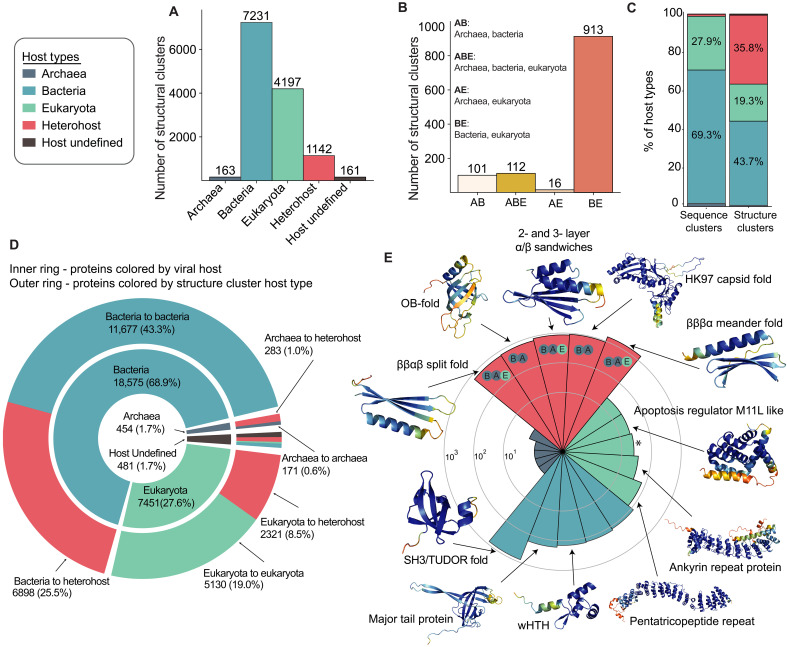
Structural clustering of VAD proteins reveals core folds in viruses infecting different domains of life. (**A**) Distribution of structural clusters by host type. (**B**) Distribution of structural heterohost clusters based on host domain composition. (**C**) Sequence and structure cluster host-type distribution of VAD proteins. (**D**) Distribution of proteins by viral host (inner ring) and structural cluster host type (outer ring), where viral host segments correspond to cluster type segments, displaying the cluster host-type membership of proteins by viral host. (**E**) Radial histogram of the five largest structural clusters, their sizes, and the structures of select cluster representatives colored by pLDDT. The contributing host taxa are shown within the heterohost segments (B, bacteria; A, archaea; E, eukaryota). The third largest eukaryote specific cluster (15 proteins; marked with an asterisk) consists of a single helix topology and is not shown.

Structural clusters show a shift in host distribution: Although bacterial host-type clusters still dominate, the proportion of eukaryotic host-type clusters increases (Fig. 3A). Heterohost clusters also expand markedly; 913 clusters include proteins from both bacterial and eukaryotic viruses, with additional clusters spanning archaea-bacteria, archaea-eukaryota, and all three domains (Fig. 3B). While fewer than 1% of VAD proteins belong to heterohost sequence clusters, this jumps to 35.8% in structural clusters (Fig. 3C). This notable increase, visualized in the network of fig. S4, suggests that folds are conserved across greater evolutionary distances and are more frequently shared across viral life than sequence similarity alone would indicate.

Next, we examined how proteins from viruses infecting specific host domains are distributed across structural clusters (Fig. 3D), as we did for sequence clusters in Fig. 1D. Most proteins from bacteriophages (62.9%) fall into clusters that also contain proteins from other bacterial viruses, but a substantial fraction (25.5%) is in heterohost clusters. Similarly, 31.1% of proteins from viruses infecting eukaryotes are in heterohost clusters, suggesting widespread structural reuse across viral lineages despite divergent host range. Archaeal viruses also show notable structural overlaps, with 62.3% of their proteins falling into archaeal-only clusters and 37.6% into heterohost clusters.

To identify common structural themes across diverse viruses, we examined the largest structural clusters in more detail (Fig. 3D). Most large structural heterohost cluster representatives are small mixed α/β folds, comprising ~4% of all proteins belonging to structural heterohost clusters (dataset S1). The largest, third largest, and fifth largest cluster representatives are α-β sandwiches: two meander folds and one split fold. The fourth largest cluster representative is an α-β OB (oligonucleotide/oligosaccharide–binding)–fold (Fig. 3E). These small folds are typical “urfolds”—three-dimensional architectures that can be formed from vastly different sequences, allowing for extensive functional innovation (*26*). The second largest structural heterohost cluster representative features an HK97 fold found in capsid proteins of various viruses. Overall, apart from the capsid fold, the largest clusters seem to represent common ancient core folds, not limited to viruses but also found in proteins across all life on Earth.

Most of the large structural eukaryotic monohost cluster representatives are α-domain structures. The two largest of these clusters are likely pentatricopeptide and ankyrin-repeat proteins, where both repeat four-helix bundles. These tandem repeat clusters comprise ~1% of all proteins belonging to structural eukaryotic clusters and ~3% of proteins belonging to nonsingleton clusters (dataset S1). The third largest cluster is a singular α helix with unstructured termini; the members of this cluster are similarly simple, with a minority exhibiting a turn between two α helices. The fourth largest of these clusters is a globular α-domain protein, the M11L-like apoptosis regulator (Fig. 3E). Thus, most of these eukaryote host–specific protein folds are likely all involved in hallmark eukaryote-like protein-protein and protein-RNA interactions (*27*, *28*). This may reflect viral adaptation to the complex and compartmentalized regulation characteristic of eukaryotic hosts (*29*).

Bacterial monohost cluster representatives consist mostly of small β-barrel folds. The largest, fourth largest, and fifth largest of these clusters have SH3/Tudor-like folds (Fig. 3E). These putative Tudor fold clusters cover ~1.3% of all proteins in bacterial structural clusters and ~2.6% of proteins belonging to nonsingleton clusters (dataset S1). The second largest cluster contains tail tube proteins from λ-like phage tail tube proteins, while the third largest cluster contains wHTH (winged helix-turn-helix) folds that are often involved in DNA binding (Fig. 3E) (*30*). Archaeal structural clusters consist almost entirely of singletons, where member proteins are uncharacterized, reflecting the paucity of data for viruses infecting this domain of life.

### Oligomerization states across viral proteomes

**Fig. 4.**
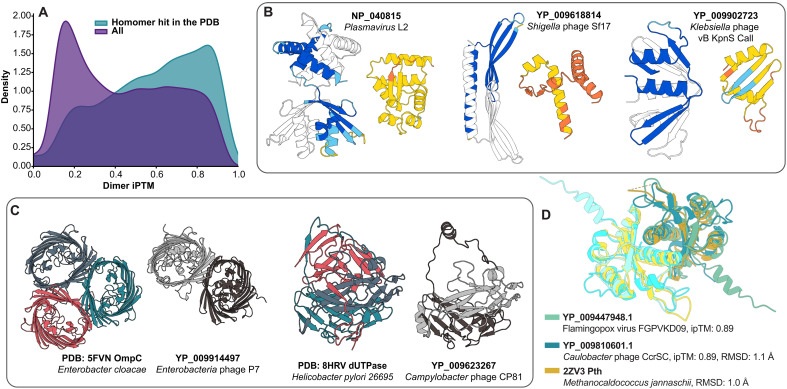
A large proportion of proteins in the VAD is predicted to form homodimeric interfaces. (**A**) Density plot of ipTM distribution for all VAD proteins and those with a homomer (homo-oligomer or homodimer) hit in the PDB. (**B**) Cases where the prediction quality markedly increases in the homodimer compared to the monomer. (**C**) Examples of cases where VAD homodimers are found in oligomer-aware conformations. Pairwise sequence identities are 65% in the case of the OmpC homologs and 28% in the case of the deoxyuridine 5′-triphosphate nucleotidohydrolases (dUTPases). (**D**) Confident dimer predictions of Pth homologs from viruses that infect both bacteria and eukaryotes. RMSD, root mean square deviation.

Many viral proteins exert their biological functions as oligomers rather than monomers. To explore oligomerization tendencies, we predicted homodimeric structures for all but the ~200 largest proteins of VAD monomers with AlphaFold-Multimer (*31*). These homodimers include 26,754 predictions, of which 11,313 exhibit a confident fold and 2957 exhibit a good fold [with interface predicted template modeling (ipTM) thresholds set to 0.5 for “confident” and to 0.8 for “good”] (Fig. 4A). Multiple VAD monomers are not predicted to form homodimers; the average ipTM score for VAD homodimers is 0.44 (dataset S1), below the cutoff for “good” models. To determine whether low-quality monomeric predictions skewed the quality of homodimer counterparts, we filtered monomers with pLDDT scores lower than 50 (fig. S5). The effect of this filtering was marginal without much change in the distribution density of predictions by the ipTM score. In 1188 cases, the pLDDT of the dimer (max across the two chains) is >5 units higher than the monomer pLDDT, with Fig. 4B showing three extreme examples, where the monomer and dimer structures have significant differences. Where we do see good scores for homodimerization, this can potentially include homodimers in the strictest sense (two monomers interacting alone), as well as larger homo- and hetero-oligomeric complexes that include an interface between two identical protein subunits. To address potentially larger complexes of VAD proteins, we predicted oligomeric arrangements of monomers in the VAD. We compared monomeric structures with those in the PDB: Structural hits of monomers found more than once in a complex in a PDB structure were classified as homo-oligomeric (3864 VAD clusters), while those found with other monomers were classified as hetero-oligomeric (2623 VAD clusters) (dataset S1). Figure 4A, indicating turquoise density, shows the dimer ipTM distribution of all VAD clusters compared to those having homomer PDB hits, showing a shift toward higher ipTM scores for the latter. AlphaFold-Multimer seems able to predict dimeric structures in their higher-order oligomeric state, as shown by the two examples in Fig. 4C where the closest Foldseek-Multimer PDB hit is a homotrimer and the predicted dimer adopts the trimeric configuration. As AlphaFold-Multimer was trained on complexes from the PDB, predicted oligomeric conformations may simply reproduce those present in the training data for close homologs. In addition, PDB structures used as templates can project oligomeric states onto homodimeric predictions. However, oligomeric awareness can also be seen at low sequence identities, as seen in the deoxyuridine 5′-triphosphate nucleotidohydrolase (dUTPase) examples in Fig. 4C, which share only 28% sequence identity.

One unexpected observation in the VAD is the prevalence of peptidyl tRNA hydrolases (Pths) across a variety of different viruses and phages. By cleaving peptides bound to tRNA, Pth plays a crucial role in protein synthesis by both rescuing stalled ribosomes and freeing up the pool of free tRNAs (*32*, *33*). We have found that Pth is encoded in various bacteriophages and viruses that infect birds and insects, and its dimeric structure is conserved (Fig. 4D). A functional Pth encoded in the bacterial host has previously been found to be important for λ phage translation of two-codon minigenes that are otherwise toxic because of accumulation of “dropped-off” peptidyl-tRNA (*34*). Thus, by carrying their own Pths, viruses may ensure efficient translation of their proteins and avoid ribosome stalling and toxicity. Our predictions suggest that the Pth sequence-level heterohost clusters are primarily homodimers. Superposition of AlphaFold Pth predictions from the *Caulobacter* phage and Flamingopox virus with the PDB structure of Pth from archaeon *Methanocaldococcus jannaschii* shows excellent structural alignment (Fig. 4D).

### Structural matches to the Protein Atlas; functional darkness and brightness of viral protein structures

**Fig. 5.**
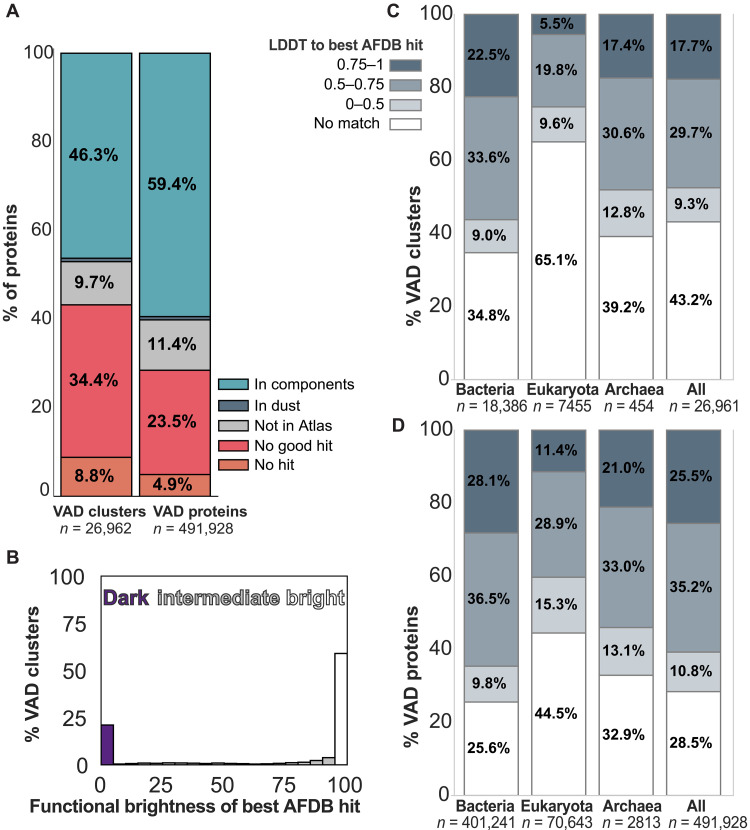
Structural similarity of VAD to the AlphaFold database. (**A**) Division of VAD representatives and proteins into those without any hits to AFDB50 or UniProt3D, without hits with TM-score >0.5, without hits to UniProt3D (i.e., only hit AFDB structures with pLDDT <90), with hits only to dust, and with hits to connected components in UniProt3D. “Representatives” mean cluster representatives that we have predicted the structure for and therefore are present in the VAD. “Proteins” extends the count to all proteins in the clusters. (**B**) Distribution of functional brightness values for the brightest AFDB hit for each VAD representative. (**C**) LDDT of best hits across different host superkingdoms as per lower inset box. (**D**) LDDT of best hits across different host superkingdoms expanded to proteins in each representative cluster.

We asked how similar or dissimilar viral proteins are to other proteins with known structures. A Foldseek search of VAD structures against UniProt3D community representatives (*14*) and the AFDB50 Foldseek database revealed 12,473 representatives (covering 292,376 viral sequences) falling into connected components in UniProt3D, 209 representatives (3635 sequences) into unconnected “dust” UniRef50 clusters, 2627 representatives (55,922 sequences) having matches to AFDB proteins not present in UniProt3D v1 because of the pLDDT threshold of 90 used, and 9279 representatives (115,750 sequences) having no good match to either database (Fig. 5A). Among those with matches to known structural communities, most are functionally bright—a term used to describe proteins with known or inferred biological function (*14*). However, a substantial fraction falls into dark communities, meaning that they lack functional annotation and remain poorly understood despite having a predicted structure (Fig. 5B). When examined by host taxonomy, proteins from viruses infecting bacteria and archaea were more likely to have high-confidence structural matches to the AFDB, whereas proteins from eukaryotic viruses had the highest proportion of no matches (Fig. 5, C and D). Among proteins that did yield a structural hit, the confidence (as measured by LDDT) was broadly similar across host types, suggesting that once a structural analog is found, the quality of the alignment does not depend strongly on host taxonomy.

### VAD proteins are related to functionally dark bacterial protein communities that include prophage-encoded TA systems

**Fig. 6.**
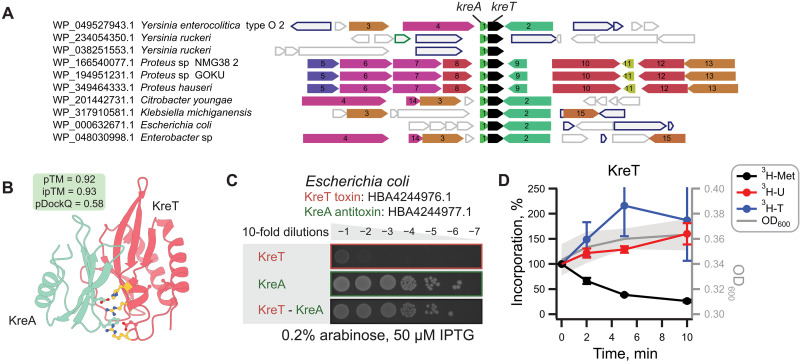
KreTA is a prophage-encoded TA system. (**A**) Gene neighborhood analysis identifies putative TA system *kreTA* encoded in prophage regions of bacterial genomes (visualized here with FlaGs). The *kreT* gene used as a FlaGs query is shown in black. Proteins encoded by the numbered genes are annotated as follows: 1: KreA; 2: recombinase/integrase; 3: major capsid protein; 4: tape measure protein; 5: deoxyribose-phosphate aldolase; 6: thymidine phosphorylase; 7: phosphopentomutase; 8: purine-nucleoside phosphorylase; 9, 13, and 14: hypothetical proteins; 10: pyridoxal-dependent decarboxylase; 11: DksA/TraR family zinc finger protein; 12: MFS transporter; 15: LysR family transcriptional regulator. (**B**) KreA and KreT are predicted to form a dimer. Putative RNase active site residues determined from (*37*) and interfacing residues are shown as sticks. Interfacing active site residues are colored yellow. (**C**) Expression of KreT is toxic to *E. coli* BW25113 cells, but the toxicity is efficiently rescued by the antitoxin KreA. (**D**) Metabolic labeling assays follow the incorporation of ^3^H-methionine (black traces), ^3^H-uridine (red), and ^3^H-thymidine (blue) upon the expression of KreT.

A previous exploration of the universe of functionally dark protein folds led to the discovery of previously unknown type II TA systems (*14*). Given that we have uncovered additional dark proteins in the VAD, we asked whether there could be further undiscovered TAs in our data. We focused on VAD proteins in Protein Atlas communities that also include bacteria, because TAs can be frequently found on prophages integrated into bacterial chromosomes (*35*, *36*). Specifically, we focused on small operon–encoded proteins encoded in communities containing *Escherichia coli*, the model system for our validations. Using GCsnap and FlaGs, we analyzed gene neighborhoods, searching for conserved bicistronic gene operons characteristic of TA systems. We found a pair with homologs across Gammaproteobacteria, consistently localized in prophage-like regions of Enterobacteriales (Fig. 6A). This includes annotated pathogenic strains isolated from clinical and food sources. The conserved two-gene architecture is strongly suggestive of a TA system. We name this putative TA system KreTA after the mythical heroic twins in the Albanian folk epic *Kângë Kreshnikësh*.

KreT and KreA proteins are predicted confidently to dimerize (Fig. 6B). The proteins are classed as functionally dark in that they belong to communities that have no functional domain hits (dataset S1) (*14*). Foldseek (*4*) also does not identify any homologous proteins with known function. However, Dali (*5*) indicates distant but significant fold similarity of KreT to ribonuclease RegB, an endoribonuclease that controls the expression of multiple phage early genes and which has no identifiable sequence similarity to anything else of known function (fig. S6A) (*37*, *38*). The closest structural relative of KreT in the VAD is annotated as RegB, but the two proteins are not confidently alignable at the sequence level. In the KreTA dimer, KreA binding sequesters the predicted ribonuclease (RNase) active site of KreT (Fig. 6B), indicative of KreA acting as an antitoxin. Furthermore, Dali suggests that KreA may have a broadly similar fold to the immunity protein Tri^Tu^ that neutralizes the adenosine 5′-diphosphate (ADP) ribosylase toxin Tre^Tu^ (fig. S6B) (*39*).

To test our prediction that KreTA is a TA, we carried out a toxicity neutralization assay. While the expression of KreT is toxic to *E. coli*, this toxicity is efficiently neutralized by the KreA antitoxin, validating that this is TA system (Fig. 6C). To investigate the mechanism of KreT toxicity, we performed metabolic labeling using radioactive precursors for translation, transcription, and replication. As manifested by the selective inhibition of ^3^H-methionine incorporation, KreT inhibits translation, which is characteristic of RNase TA toxins (Fig. 6D) (*40*). Together, the fold similarity of KreT to endonucleases and its inhibition of translation suggest that it may function by cleaving mRNA, as observed for many known TA toxins (*40*). While KreT and KreA are viral in origin, being encoded on prophages, there are no relatives of either in the VAD that are identifiable at the sequence level. This highlights another challenge for understanding viral proteome diversity: that viruses can go under the radar by hiding within the genome of their hosts. While “cryptic” infections are most well known for phages, this is even the case for some eukaryotic viruses (*41*, *42*).

### Conserved enzymatic folds are co-opted in antiviral defense and counterdefense

Viruses are territorial; when they have infected a host cell, it is in their interest to keep out competitor viruses. This is called superinfection exclusion and is observed in viruses that infect bacteria as well as eukaryotes (*43*, *44*). Temperate bacteriophages have been found to carry a rich diversity of phage defense systems (*45*). To analyze defense-like folds in the VAD, we used Foldseek to search our structures against the DefenseFinder database (*46*).

**Fig. 7.**
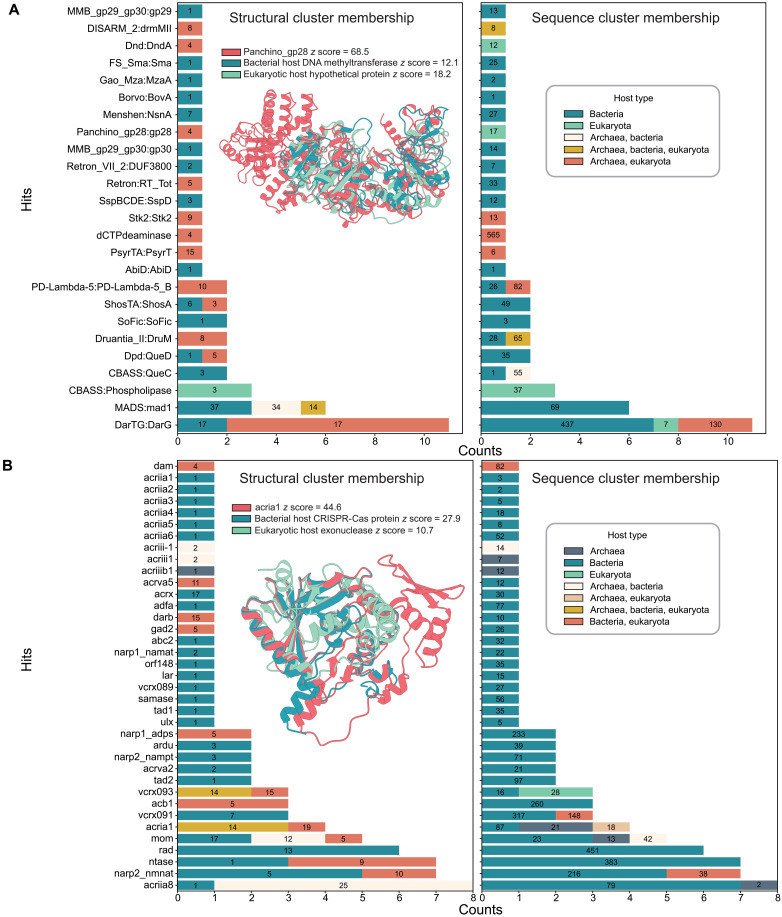
Antiphage defense–related proteins in the VAD. Hits are colored by structural cluster host type and numbered by structural cluster size (left) and by sequence cluster host type and sequence cluster size (right). Each panel includes an example defense system protein, a VAD hit aligned to it, and a protein from a different host type within the same structural cluster, aligned using Dali. (**A**) DefenseFinder structural hits, with the example alignment showing Panchino_gp28 (*z* score = 68.5), a VAD hit (eukaryotic host hypothetical protein; *z* score = 18.2), and a different host type protein (bacterial host DNA methyltransferase; *z* score = 12.1). Numbers on bars refer to the size of the cluster that the protein with a hit to a defense protein belongs to. For example, DarG has 11 hits to two different cluster host types, each of which has a size of 17. (**B**) Combined structural hits from AntiDefenseFinder and dbAPIS, with the example alignment showing AcrIA1 (*z* score = 44.6), a VAD hit (phage CRISPR-Cas protein; *z* score = 27.9), and a different host type protein [viral exonuclease (eukaryotic host); *z* score = 10.7].

The largest number of hits to one defense system protein is for the DarG protein from the DarTG system with nine hits to the same heterohost structural cluster with a eukaryotic host-type representative (Fig. 7A; pink bar). Proteins in this cluster are all annotated as macrodomain or ADP-ribosyl glycohydrolase proteins (dataset S1). The alignments of these proteins to DarG align solely to their macrodomain and not to the C-terminal region implicated in binding DarT (fig. S7) (*47*). These hits may actually be counterdefense-related via the reversal of host ADP-ribosylation catalyzed in eukaryotic antiviral responses (*48*). However, given that macrodomains have multiple functions, we cannot rule out other roles beyond defense or counterdefense. Notably, six of the structural heterohost cluster proteins with hits to DarG belong to sequence monohost clusters, indicating a conservation of macrodomain folds across viruses infecting different domains of life, even when sequence similarity is not apparent (Fig. 7A).

Another notable hit to defense proteins is that of gp28 in the Panchino system. This protein contains restriction, modification, and specificity domains of type I restriction modification proteins (*49*). The VAD protein, which matches Panchino_gp28, is a hypothetical protein belonging to a heterohost structural cluster with a eukaryotic host-type representative. Within this cluster, the best-aligning protein is a bacterial host-type methyltransferase (Fig. 7A and dataset S1). Proteins in this cluster all have PDB hits to methyltransferase proteins (dataset S1). The alignments to Panchino_gp28 cover mostly the methyltransferase domain but also extend to the restriction domain (Fig. 7A). As before with DarG, the VAD protein with a hit to Panchino_gp28 belongs to a sequence monohost cluster and a structural heterohost cluster, providing another example of cross-domain conservation of folds not apparent on the sequence level (Fig. 7A).

As well as defending against superinfection, viruses carry counterdefense systems. To investigate whether counterdefense folds are found in the VAD, we searched against antidefense proteins in the DefenseFinder database (*46*) and dbAPIS (*50*). The antidefense protein with the most hits to VAD proteins is AcrIIA8 (Fig. 7B), a phage head-tail adapter protein that—unexpectedly—is implicated in antidefense by inhibiting Cas9 activity (*51*). AcrIIA8 has seven hits within the same structural cluster containing bacterial and archaeal host-type proteins, along with one hit to a bacterial host-type singleton cluster (Fig. 7B). When clustered by sequence, AcrIIA8 hits belong to either archaeal or bacterial host-type clusters, and these clusters merge at the structure level, reflecting a conserved fold across host types (Fig. 7B). Proteins in these clusters are similarly annotated as phage head, neck, and tail structural proteins, and it is unclear whether they could also have additional moonlighting antidefense functions (dataset S1).

AcrIA1, an anti-CRISPR exonuclease affecting spacer acquisition in CRISPR-Cas–mediated defense (*52*), has hits to VAD proteins belonging to sequence and structure heterohost clusters (Fig. 7B). Structural representatives of these clusters are annotated as CRISPR-Cas associated and/or exonucleases, including a PD-(D/E)XK superfamily nuclease (dataset S1) (*53*). AcrIA1 aligns well with its bacterial host-type CRISPR-Cas–associated hit, as well as a eukaryote-host exonuclease found in the same structural cluster (Fig. 7B). This eukaryote-host exonuclease is remarkably similar in both sequence and structure to phage exonucleases (dataset S1). Furthermore, it has a hit to another CRISPR-Cas–associated antidefense protein, VCRX093 (Fig. 7B and dataset S1) (*54*). Given that eukaryotes do not carry CRISPR-Cas immune systems, this nuclease likely has an as-yet-undefined role in nucleic acid cleavage during the viral life cycle. In addition, its Foldseek hits to R354—a Cas4-like nuclease from the MIMIVIRE virophage resistance system in mimiviruses (*55*)—raise the possibility that it may represent a functionally analogous, virophage defense or genome processing element.

Last, phosphodiesterases have been found to be conserved across viruses that infect bacteria and eukaryotes (*10*). This class of proteins includes the antidefense protein anti-CBASS protein (Acb1), which degrades cyclic nucleotides (*11*, *56*). Acb1 also has hits to VAD proteins belonging to a structural heterohost cluster (Fig. 7B), further supporting its functional role in cross-domain counterdefense.

## DISCUSSION

Comprising high-quality structural predictions of monomers and dimers, the VAD provides a resource for exploring viral diversity, evolution, and biology. There are significant functional “darkness” within viral proteomes and much novel biology awaiting discovery. Through searching for similarities of viral protein structures to communities of proteins in the dark corners of the protein universe, we have uncovered a previously unidentified Enterobacteriales prophage–encoded TA system, KreTA.

Our large-scale structural analysis reveals that despite vast sequence diversity, viruses often rely on conserved protein folds that cross host boundaries and recur across the tree of life. The structural similarities between viral proteins and defense-related systems highlight the repeated co-option of enzymatic folds in microbial conflicts. The observed conservation in viral defense and counterdefense systems is primarily confined to relatively large enzymes such as methyltransferases, ADP-ribose–processing macrodomains, and exonucleases. In contrast, small inhibitors, which are particularly common in counterdefense, appear to be less conserved. However, it is important to note that small proteins are inherently more difficult to detect confidently in structural homology searches, especially if their function depends on—and the structure changes upon—oligomerization. Overall, while viruses use diverse strategies to evade or counteract host defenses, the structural folds of enzymatic proteins involved in these processes are more likely to be preserved—or at least detectably conserved—across different domains of life. Notably, these conserved enzymatic folds are not unique to viruses but are repurposed from universal protein architectures that span across bacteria, archaea, and eukaryota. This structural conservation highlights the reuse of molecular mechanisms in host-virus interactions, underscoring that core and likely ancient protein folds remain essential components of defense and counterdefense systems. Together, our findings show that conserved structural frameworks underpin key functions in infection, defense, and adaptation across all domains of life.

## MATERIALS AND METHODS

### Sequence acquisition and host assignment of proteins

A total of 647,000 virus protein sequences was downloaded from the NCBI RefSeq (*20*) database (ncbi.nlm.nih.gov; April 2023). NCBI records for each protein sequence in the RefSeq virus dataset are used to attain taxonomy IDs for the virus the protein is found in. These IDs are then matched to entries in the Virus-Host database (*21*) to assign a host lineage to each protein. Approximately 10,000 RefSeq virus records lack taxonomy IDs, and about 3000 virus taxonomy IDs are not found in the Virus-Host database. Proteins associated with these missing or unrecognized taxonomy IDs, as well as those from viruses that infect other viruses and proteins without assigned hosts in the Virus-Host database, are labeled as “Host undefined.” While these proteins are included in the dataset, they do not contribute to host-type classifications. Through manual curation, certain Virus-Host database hosts were determined to be wrongly or insufficiently classified. Viral lineages *Flyfo siphovirus Tbat1_6*, *uncultured phage*, and *Leviviridae* sp. were set to bacteria-infecting. Viral lineages *Dunaliella viridis virus* and *Tetraselmis viridis virus* were set to “Host undefined.”

### Sequence clustering and host assignment of clusters

MMSeqs2 (version 15.6f452) (*22*) was used to cluster RefSeq viral sequences and select representatives for structural analysis. The sequence identity threshold was set to 30%, and the query to target coverage threshold was set to 10%. This resulted in 117,479 clusters with 61,868 singletons. The clusters were assigned a host type on the basis of the host types of the proteins within them. To limit the effect of possible isolated cases of virus-host misannotation on heterohost assignment, we set a minimum threshold on the number of mixed hosts in order for a cluster to be assigned a heterohost label. We required that at least three proteins or 1% of the total cluster size (whichever is bigger) be of different host origin. The remaining clusters are classified as archaea, bacteria, or eukaryota monohost clusters. Only clusters composed solely of proteins without a defined host are classified as “Host undefined.”

### Structural prediction of monomers and homodimers

Protein structure predictions were made with AlphaFold2 version 2.3.2 (*1*) with default parameters. Homodimers were predicted with the AlphaFold-Multimer protocol (*31*). The structural template cutoff date was set to 14 May 2020 (--max_template_date = 2020-05-14). Sixty-three sequence cluster representatives were not predicted because of out-of-memory and database asynchronicity errors in the AlphaFold2 pipeline.

The quality of structural predictions was assessed with pLDDT (*1*) scores for monomers and predicted template modeling (pTM + ipTM) (*31*) and predicted DockQ (pDockQ) (*57*) scores for homodimers. The best models, determined by pLDDT scores for monomers and pTM + ipTM scores for homodimers, were selected for further analysis. Foldseek (*4*) exhaustive search without thresholds was used to perform all-versus-all alignments of VAD monomers.

### Structural clustering and host assignment of clusters

The VAD was structurally clustered with Foldseek (version 8.ef4e960) (*4*) in TMalign mode. Threshold parameters for the clustering were set at 90% for alignment coverage, 0.4 for the template modeling score (TM-score), and 0.001 for the *E*-value. Structural clusters were assigned a host type as was done with sequence clusters.

### Searches against other databases

VAD structures and Foldseek (*4*) were used to search against the following structural databases: AFDB (*13*), PDB (*12*), and UniProt3D community representatives (*14*). The Dali webserver (*58*) was also used to search the PDB (*12*).

### Genomic context analysis

For the 871 viral protein clusters belonging to prokaryotic hosts (i.e., bacteria, archaea, and bacteria and archaea) with a match to a dark community in the Protein Universe Atlas network (i.e., median functional brightness below 5%) with at least five protein members, genomic context analysis was conducted using GCsnap (*59*) with default parameters. For the 2313 tested Protein Universe Atlas community (*14*), all UniProt IDs linked to that community were used as input targets. To aid the visual inspection of gene neighborhoods, FlaGs2 (*60*) was also used.

Conserved genomic windows identified by GCsnap were analyzed for the distribution of protein family names (as automatically assigned by GCsnap) around viral protein targets. To uncover communities containing conserved bicistronic gene arrangements with characteristics of TA systems, genomic contexts containing target proteins from at least six different species were systematically screened. This approach was based on criteria adapted from NetFlax (*35*) and further generalized through the analysis of GCsnap outputs across diverse datasets. The gene pattern prediction relied on the following criteria: (i) Genes within a pattern must appear in the same order; (ii) intergenic distances between genes within the pattern should not exceed 100 nucleotides, while distances to conserved genes outside the pattern should exceed 100 nucleotides; (iii) neighborhood conservation should be restricted to the genes within the pattern; and (iv) the relative frequency difference between a pattern and the most frequent longer variant (i.e., one additional gene) should be maximal within the genomic context. This screening approach identified the most promising dark communities potentially harboring TA systems, providing a focused set of candidates for further inspection and functional analysis.

### Predicting higher-order oligostates

Oligomeric states for PDB structures were obtained from the Swiss-Model Template Library (*61*). To predict the oligomeric state of a VAD cluster protein, we ran a Foldseek search against all chains in the PDB with a TM-score threshold of 0.5 and returned the most common oligomeric state of all the hits found weighted by the TM-score.

### Searching against defense and antidefense databases

DefenseFinder (*46*) models were downloaded from https://defensefinder.mdmlab.fr/wiki/structure on 16 January 2025. For each defense system protein, one monomeric model was used in analyses. Antidefense protein hidden Markov models were manually downloaded from https://github.com/mdmparis/defense-finder-models (commit ca8f119). The PyHMMER (version 0.10.15) (*62*) function most_probable_sequence was used to extract sequences from hidden Markov models. AlphaFold2 (*1*) version 2.3.2 was used to model sequences extracted with the same runtime parameters as VAD structure prediction. This dataset is referred to as AntiDefenseFinder. dbAPIS (*50*) models were downloaded from https://bcb.unl.edu/dbAPIS/downloads/ on 9 February 2024. Foldseek search without prefiltering and with a TM-score threshold of 0.5 was used to compare VAD structures to DefenseFinder, AntiDefenseFinder, and dbAPIS models. For each VAD protein, the hit with the best identity fraction time query coverage was kept. After exhaustive searching, further filters were applied when plotting hits. DefenseFinder: alignment TM-score >0.65, *E*-value <0.001, average alignment coverage >0.6, cluster average TM-score >0.5. AntiDefenseFinder + dbAPIS: alignment TM-score >0.65, *E*-value <0.001 (for AntiDefenseFinder only), average alignment coverage >0.5, cluster average TM-score >0.5, intracluster alignment length >100. In addition, when plotting, AntiDefenseFinder and dbAPIS hits were combined where the hit with the best TM-score per VAD protein was kept.

### Construction of plasmids

All primer and plasmid map designs were generated using SnapGene (GSL Biotech LLC) and Geneious Prime (Biomatters). All plasmids were constructed using circular polymerase extension cloning (*63*, *64*) using Phusion High-Fidelity DNA Polymerase (Thermo Fisher Scientific). Detailed cloning strategies, including primer sequences and assembly schemes, are provided in table S3.

### TA neutralization assays

The experiments were performed as described earlier, with minor modifications (*14*). The genes encoding the candidate toxin *kreT* (WP_000632671.1) and candidate antitoxin *kreA* (WP_ 001008346.1) were cloned into pBAD33 and pMG25 backbones, respectively, yielding VHP1906 (*kreT*) and VHP1970 (*kreA*) plasmids. *E. coli* BW25113 cells were cotransformed either with the plasmid pair expressing the TA system in trans or with one of the two protein-expressing plasmids being swapped for the appropriate empty vector. The cells were grown for 5 hours at 37°C with shaking at 200 rpm in an LB medium supplemented with carbenicillin (100 μg/ml; to maintain pMG25 and its derivatives), chloramphenicol (25 μg/ml; to maintain pBAD33 and its derivatives), and 0.2% glucose for the repression of the toxin expression. Next, the cells were diluted in an LB medium to a final optical density at 600 nm (OD_600_) of 1.0, and serial 10-fold dilutions (from 10^−1^ to 10^−7^) were made in LB. The dilutions were spotted on LB agar plates supplemented with carbenicillin (100 μg/ml), chloramphenicol (25 μg/ml), 50 μM IPTG (isopropyl-β-d-thiogalactopyranoside; for antitoxin expression), and 0.2% arabinose (for toxin expression). The plates were scored after an overnight incubation at 37°C.

### Metabolic labeling assays

The labeling assays were also performed as described earlier, with minor modifications (*14*). A single colony of *E. coli* BW25113 cells expressing the KreT toxin from the pBAD33 derivative plasmid under the control of arabinose-inducible P*_BAD_* promoter (VHP1976) was grown overnight in 2 ml of Neidhardt Mops minimal medium (*65*) supplemented with 1% glucose, 0.1% casein hydrolysate, and chloramphenicol (25 μg/ml) at 37°C with shaking at 160 rpm in MaxQ 6000 Shaker (Thermo Fisher Scientific) . The overnight culture was used to inoculate a 20-ml culture in MOPS minimal medium supplemented with 0.5% glycerol as well as a set of 19 amino acids lacking methionine (each at 25 μg/ml) to the final OD_600_ of 0.05.

The cells were grown in a 100-ml flask at 37°C with shaking (160 rpm) in OLS Aqua Pro Shaking Water Bath (Grant Instruments) until recovery after the diauxic shift was observed (OD_600_ ~ 0.2 to 0.3). At this moment, 1-ml zero-time-point aliquots were taken and combined in 1.5-ml sterile Eppendorf tubes with either ^3^H-thymidine (PerkinElmer) (10 μl per point, 4.5 μCi “hot” and 4.8 μM “cold” nucleotides in autoclaved distilled water), ^3^H-uridine (PerkinElmer) (10 μl per point, 0.56 μCi “hot” and 4.97 μM “cold” nucleotides in autoclaved distilled water), or ^3^H-methionine (Revvity) (10 μl per point, 0.67 μCi “hot” and 14.99 μM “cold” amino acids in autoclaved distilled water). After an 8-min incubation at 37°C, the zero-time-point labeling reactions were quenched by the addition of 200 μl of ice-cold 50% trichloroacetic acid (TCA) and transferred on ice.

Immediately after the collection of the zero time, the KreT expression was induced by adding l-arabinose to the culture to a final concentration of 0.2%. At 2, 5, 10, and 15 min postinduction, 1-ml culture aliquots were taken, combined with the “hot” label and processed analogously to the zero-time-point samples. OD_600_ values were recorded for each time point. The TCA-quenched samples were filtered through GF/C filters (Whatman) prewashed with 5 ml of 5% TCA. The filters were washed with 5 ml (twice for ^3^H-methionine and ^3^H-uridine and three times for ^3^H-thymidine) of ice-cold 5% TCA and then with 95% ice-cold ethanol (5-ml washes, twice for ^3^H-methionine and ^3^H-uridine and three times for ^3^H-thymidine). The filters were placed in 20-ml scintillation vials (Sarstedt) and air dried until they were dry (at least for 2 hours) at room temperature. Five milliliters of EcoLite Liquid (MP Biomedicals) scintillation cocktail was added to each vial, followed by shaking for 15 min before counting. The radioactivity was quantified in CPM (counts per minute) using a Hidex 600 SLe automatic liquid scintillation counter (Hidex). CPM values were normalized to OD_600_ at each time point, and incorporation percentages were calculated by dividing the normalized CPM/OD_600_ values by the corresponding value at zero-time-point values. All experiments were performed using three independent biological replicates and presented as the mean values ± SD.

### Data visualization

The VAD network was built using NetworkX (version 3.4.2) (*66*). The network was drawn and visualized with Gephi (version 0.10.0) (*67*). Structures were visualized and superimposed with Chimera (*68*), and metabolic labeling data were visualized using Igor Pro 7 (WaveMetrics). The Pavian package (*69*) was used to plot Sankey diagrams. The VAD network was built using NetworkX (version 3.4.2), drawn and visualized with Gephi, and deployed with sigma.js. Web application protein structures are displayed using Mol* (*70*).
