# Supplementary material for: The Viral AlphaFold Database of monomers and homodimers reveals conserved protein folds in viruses of bacteria, archaea, and eukaryotes
Source: Sci Adv. 2025 Oct 1;11(40):eadz8560. doi: 10.1126/sciadv.adz8560 (PMC12487901; doi:10.1126/sciadv.adz8560)
Supplement: Supplementary file 1 — Figs. S1 to S7 Tables S1 and S2 Legend for table S3 Legend for dataset S1 [file sciadv.adz8560_sm.pdf]

Science Advances
AAAS

Supplementary Materials for

The Viral AlphaFold Database of monomers and homodimers reveals conserved protein folds in viruses of bacteria, archaea, and eukaryotes

Roni Odai *et al.*

Corresponding author: Vasili Hauryliuk, vasili.hauryliuk@med.lu.se; Janani Durairaj, janani.durairaj@unibas.ch; Joana Pereira, joana.pereira@kuleuven.be; Gemma C. Atkinson, gemma.atkinson@med.lu.se

Sci. Adv. **11**, eadz8560 (2025)
DOI: 10.1126/sciadv.adz8560

The PDF file includes:

Figs. S1 to S7
Tables S1 and S2
Legend for table S3
Legend for dataset S1

Other Supplementary Material for this manuscript includes the following:

Table S3
Dataset S1

Supplementary Figures

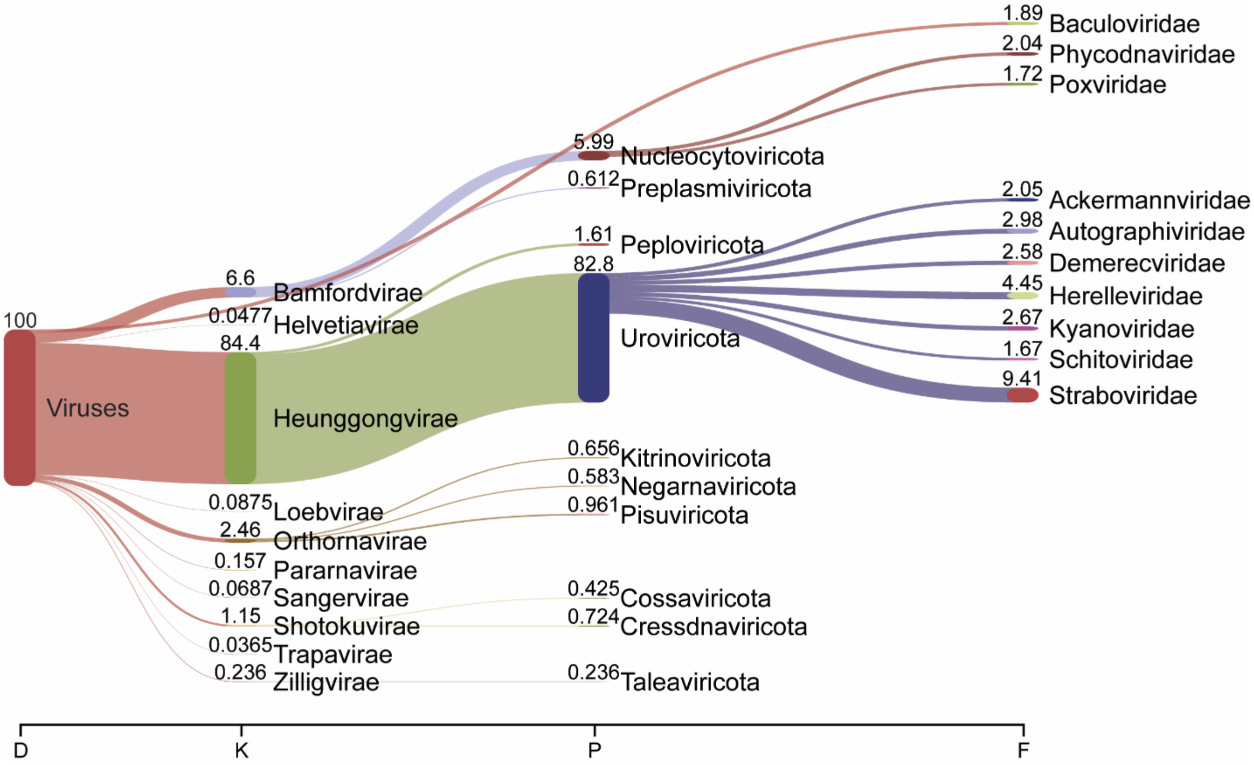

fig. S1. Taxonomic distribution of RefSeq viral sequences.
The ten most abundant taxa per ranks Domain (D), Kingdom (K), phylum (P), family (F), and their relative abundance are shown.

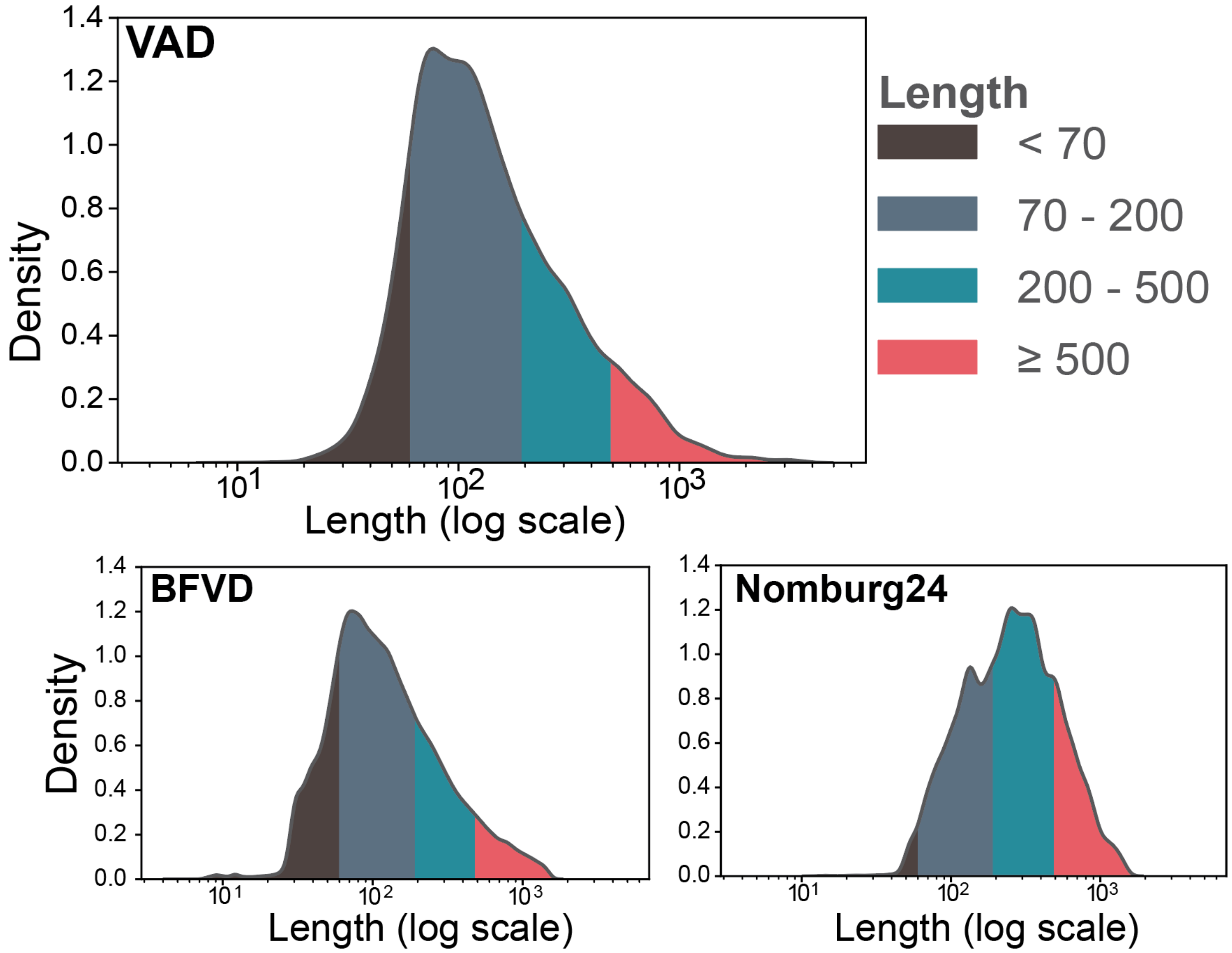

fig. S2. VAD length distribution in comparison with other databases of viral predicted structures. Length distributions for VAD, BFVD, and Nomburg24 viral protein structure databases.

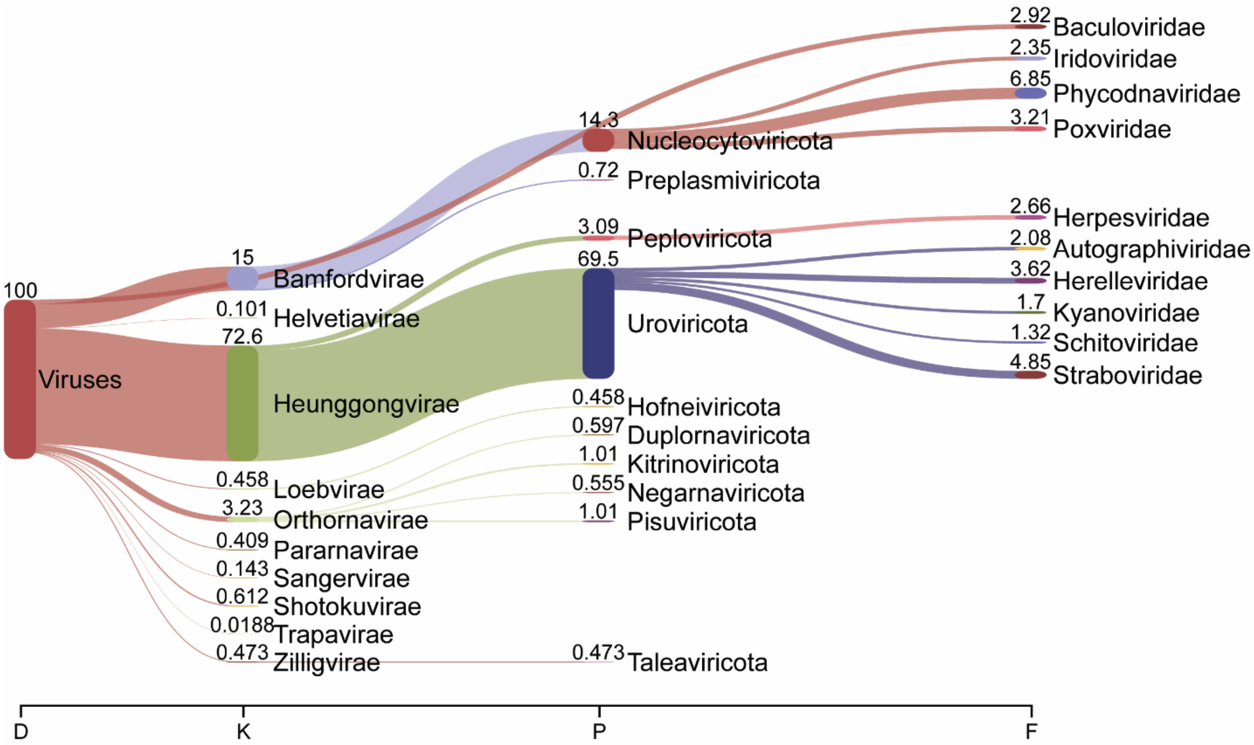

fig S3. Taxonomic distribution of VAD.
The ten most abundant taxa per ranks Domain (D), Kingdom (K), phylum (P), family (F), and their relative abundance are shown.

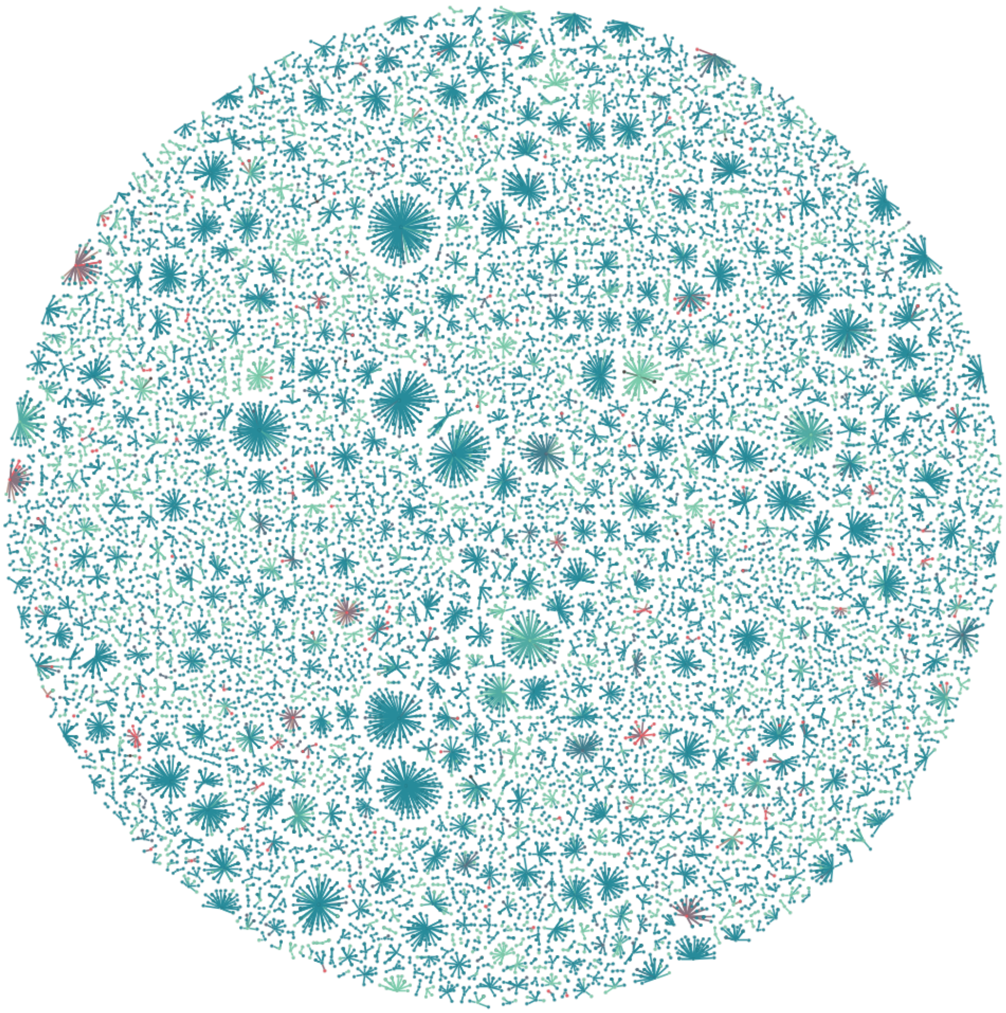

fig S4. VAD network of sequence clusters joined into structural alignment clusters.
Nodes represent individual VAD proteins (sequence cluster representatives), with edges drawn between each cluster representative and its corresponding members. Node and edge colors indicate the host type associated with each protein's sequence cluster. Singletons are removed; only structural clusters of two or more are considered. An interactive version of the network is available at vad.atkinsonlab.com.

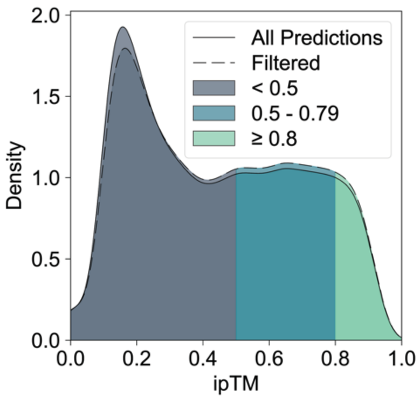

fig S5. The ipTM distribution of VAD homodimers does not substantially change when filtered by monomer quality.

Density plot of ipTM Distribution. Dimeric predictions are filtered based on the average pLDDT score of their monomeric counterparts, removing those with an average pLDDT below 50.

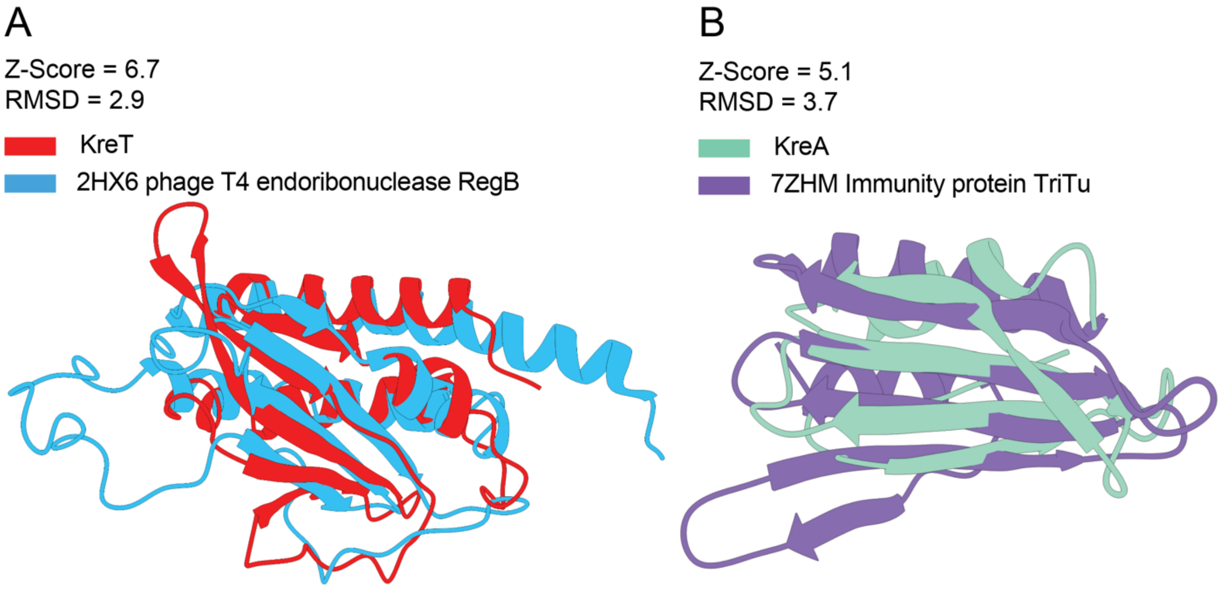

fig. S6. Structural search supports KreTA as a novel toxin-antitoxin system.
(**A**) KreT is likely an endoribonuclease toxin. DALI alignment reveals structural similarity to the phage T4 endoribonuclease RegB (PDB ID: 2HX6), suggesting a potential role in RNA cleavage.
(**B**) KreA shows structural similarity to the immunity protein TriTu (PDB ID: 7ZHM), an antitoxin, supporting its annotation as the antitoxin partner in the KreTA system.

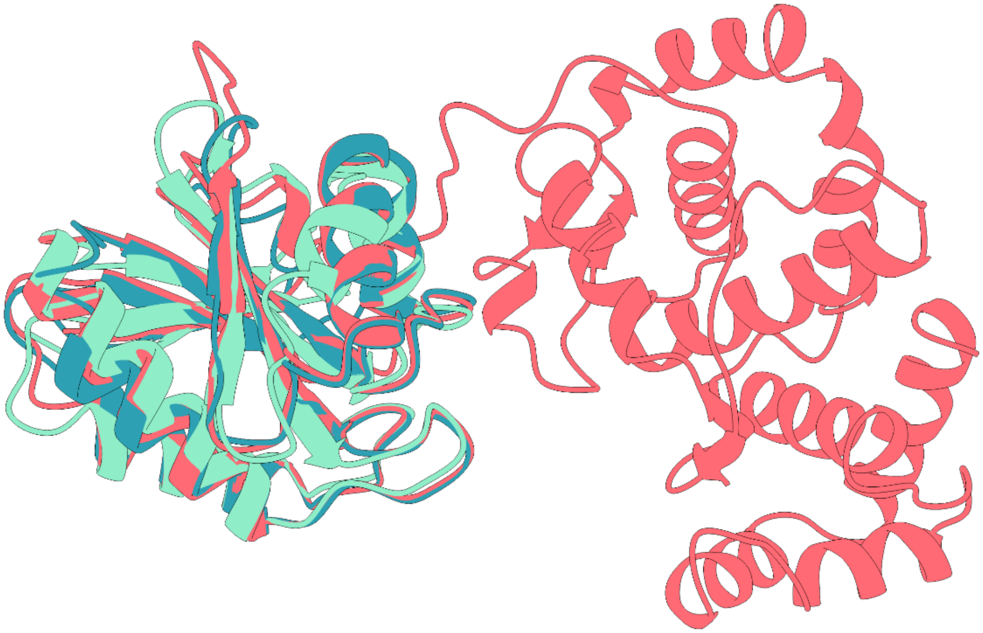

fig. S7. DarG partial alignments to VAD proteins, covering macrodomain.
DarG (red) aligns with eukaryotic host-type (green; YP_009163851.1) and bacterial host-type (blue; YP_010644491.1) VAD proteins within the same structural cluster, showing partial alignment to the N-terminal macrodomain of DarG. Both VAD proteins are hits to DarG.

Supplementary tables

table S1. Sequence cluster sizes

Sequence clusters	Amount of clusters	Amount of singletons	Amount of Proteins	Average cluster size	Standard deviation of cluster size
Overall	117,479	61,868	647,000	5.5	18.4
Archaea	3,465	2,243	7,061	2.0	2.3
Bacteria	86,130	42,331	521,229	6.1	19.0
Eukaryota	25,270	15,314	99,476	4.0	14.4
Hetero Host	388	0	16,590	42.8	78.5
Host Undefined	2,226	1,980	26,44	1.2	0.7

table S2. Structure cluster sizes

Structural clusters	Amount of clusters	Amount of singletons	Amount of Proteins	Average cluster size	Standard deviation of cluster size
Overall	12,894	9,753	26,962	2.1	4.1
Archaea	163	156	171	1.0	0.2
Bacteria	7,231	5,720	11,788	1.6	2.3
Eukaryota	4,197	3,718	5,190	1.2	1.0
Hetero Host	1,142	0	9,650	8.5	10.5
Host Undefined	161	159	163	1.0	0.1

table S3 (separate file).
Information is listed in separate tabs on on plasmids, cloning strategy, primers, DNA fragments and strains.

dataset S1 (separate file).
This file is available from doi.org/10.6084/m9.figshare.29835206. Additional VAD metadata is organised in tabs: taxonomic lineages of source genomes, cluster composition, host assignments, and Foldseek hit information to defense and counter defense databases.
